# Timely intervention and control of a novel coronavirus (COVID-19) outbreak at a large skilled nursing facility—San Francisco, California, 2020

**DOI:** 10.1017/ice.2020.1375

**Published:** 2020-12-14

**Authors:** Ellora N. Karmarkar, Irin Blanco, Pauli N. Amornkul, Amie DuBois, Xianding Deng, Patrick K. Moonan, Beth L. Rubenstein, David A. Miller, Idamae Kennedy, Jennifer Yu, Justin P. Dauterman, Melissa Ongpin, Wilmie Hathaway, Lisa Hoo, Stephanie Trammell, Ejovwoke F. Dosunmu, Guixia Yu, Zenith Khwaja, Wendy Lu, Nawzaneen Z. Talai, Seema Jain, Janice K. Louie, Susan S. Philip, Scot Federman, Godfred Masinde, Debra A. Wadford, Naveena Bobba, Juliet Stoltey, Adrian Smith, Erin Epson, Charles Y. Chiu, Ayanna S. Bennett, Amber M. Vasquez, Troy Williams

**Affiliations:** 1 Epidemic Intelligence Service Program, Division of Scientific Education and Professional Development, Centers for Disease Control and Prevention, Atlanta, Georgia; 2 California Department of Public Health, Richmond, California; 3 Laguna Honda Hospital, San Francisco, California; 4 San Francisco Department of Public Health, San Francisco, California; 5 University of California, San Francisco, San Francisco, California; 6 Division of Global HIV and Tuberculosis, Centers for Disease Control and Prevention, Atlanta, Georgia; 7 San Francisco Public Health Laboratory, San Francisco, California; 8 Viral and Rickettsial Disease Laboratory, California Department of Public Health, Richmond, California; 9 Division of Healthcare Quality Promotion, Centers for Disease Control and Prevention, Atlanta, Georgia

## Abstract

**Objective:**

To describe epidemiologic and genomic characteristics of a severe acute respiratory syndrome coronavirus 2 (SARS-CoV-2) outbreak in a large skilled-nursing facility (SNF), and the strategies that controlled transmission.

**Design, setting, and participants:**

This cohort study was conducted during March 22–May 4, 2020, among all staff and residents at a 780-bed SNF in San Francisco, California.

**Methods:**

Contact tracing and symptom screening guided targeted testing of staff and residents; respiratory specimens were also collected through serial point prevalence surveys (PPSs) in units with confirmed cases. Cases were confirmed by real-time reverse transcription–polymerase chain reaction testing for SARS-CoV-2, and whole-genome sequencing (WGS) was used to characterize viral isolate lineages and relatedness. Infection prevention and control (IPC) interventions included restricting from work any staff who had close contact with a confirmed case; restricting movement between units; implementing surgical face masking facility-wide; and the use of recommended PPE (ie, isolation gown, gloves, N95 respirator and eye protection) for clinical interactions in units with confirmed cases.

**Results:**

Of 725 staff and residents tested through targeted testing and serial PPSs, 21 (3%) were SARS-CoV-2 positive: 16 (76%) staff and 5 (24%) residents. Fifteen cases (71%) were linked to a single unit. Targeted testing identified 17 cases (81%), and PPSs identified 4 cases (19%). Most cases (71%) were identified before IPC interventions could be implemented. WGS was performed on SARS-CoV-2 isolates from 4 staff and 4 residents: 5 were of Santa Clara County lineage and the 3 others were distinct lineages.

**Conclusions:**

Early implementation of targeted testing, serial PPSs, and multimodal IPC interventions limited SARS-CoV-2 transmission within the SNF.

Skilled nursing facility (SNF) staff care for medically fragile residents, often in settings with limited infection prevention and control (IPC) capacity.^1,2^ Given the substantial morbidity and mortality during novel coronavirus disease 2019 (COVID-19) outbreaks in SNFs nationwide, SNFs are a high priority for outbreak prevention and control.^3–6^ Multiple studies describe high attack rates of severe acute respiratory coronavirus 2 (SARS-CoV-2) within SNFs, often through asymptomatic and pre-symptomatic transmission occurring prior to implementation of IPC interventions.^4,5,7^ With continued community-based SARS-CoV-2 transmission nationwide,^8^ staff pose an ongoing risk of introducing the virus to SNFs.^9^ Mass testing of all staff and residents in SNFs for SARS-CoV-2 is recommended.^5,10,11^ However, limited testing and personal protective equipment (PPE)^6^ resources early in the pandemic impaired effective implementation of mass testing and transmission-based precautions. Including robust IPC measures with testing is essential to successfully interrupting transmission.^7,12^

This study was conducted in a 780-bed (23 single rooms, 7 airborne infection isolation rooms, 195 double rooms, and 120 triple rooms) SNF and rehabilitation center with 2 towers and a pavilion, affiliated with the San Francisco Department of Public Health (SFDPH). It is one of the largest SNFs in the United States, with >1,700 staff, 13 specialized SNF units, and 1 acute-care unit. There is 1 designated IPC staff member for the entire facility.

On March 7, 2020, SFDPH issued a health order restricting all visitors, vendors, and volunteers from entering the facility, in response to the COVID-19 pandemic. The facility still permitted residents to congregate in the common areas on their units.

On March 22, 2020, a symptomatic staff member in unit A tested positive for SARS-CoV-2 by real-time reverse transcription-polymerase chain reaction (rRT-PCR). By March 25, 4 additional symptomatic staff tested positive for SARS-CoV-2 (2 on unit A and 2 on unit B). The national testing and PPE shortages at the time and the number of residents (718) and staff (1,704) on site precluded facility-wide testing. To prevent a potentially catastrophic outbreak, the facility and SFDPH collaborated with the California Department of Public Health (CDPH) and Centers for Disease Control and Prevention (CDC) to rapidly identify cases among residents and staff, to intensify IPC interventions, and to monitor facility-based transmission with a resource-conscious approach.

## Methods

This investigation was a public health response; data collection was determined to be non–human-subjects research by the CDC.

### Case finding

A case was defined as a laboratory-confirmed SARS-CoV-2 infection in a resident or staff member, detected by positive nasopharyngeal swab through rRT-PCR. Beginning March 22, a contact-tracing team comprising public health staff and facility administration interviewed staff with laboratory-confirmed SARS-CoV-2 and reviewed staffing logs and medical charts to identify close contacts. A close contact was defined as a person who spent ≥15 minutes within 6 feet of a person with laboratory-confirmed SARS-CoV-2 infection. All staff identified as close contacts were placed on sick leave, instructed to quarantine and self-monitor for symptoms for 14 days at home, and advised to seek testing. Symptomatic close contacts were prioritized for immediate testing by their private provider or the facility.

Beginning March 23, all staff were screened daily in their assigned unit during their work shift for symptoms consistent with COVID-19,^13^ including fever, cough, sore throat, loss of taste or smell, and shortness of breath. On April 2, to identify ill staff before building entry, screening was transitioned to 2 main facility entrances. Unit A and B staff had additional mid-shift screening on their assigned unit. Symptomatic staff could not enter the building or continue their work day; they were sent home on sick leave, followed up by phone, and referred for required testing prior to returning for work.

In addition to symptom screening, staff were encouraged to report symptoms or close contact with any person with laboratory-confirmed SARS-CoV-2 infection. Staff calling in sick were contacted and screened for COVID-19 symptoms. Symptomatic staff were referred for testing and were instructed to self-isolate for at least 7 days and until complete symptom resolution regardless of test results. Staff with laboratory-confirmed SARS-CoV-2 could not return to work until all of the following occurred: (1) symptom resolution, (2) at least 7 days since symptom onset and at least 72 hours since symptom improvement, and (3) 2 negative SARS-CoV-2 rRT-PCR respiratory tests separated by >24 hours.

Facility-wide, residents were screened at least daily for symptoms (including mental status changes) and abnormal vital signs. Any resident with new symptoms or vital-sign changes was tested for SARS-CoV-2, but no more frequently than once every 3 days. On units A and B, residents were screened every 8 hours. Facility-wide, any resident with a recent hospitalization or regular specialized care outside the facility (eg, infusions, dialysis) was screened every 8 hours; recently hospitalized residents were screened for 14 days and residents receiving specialized care were screened indefinitely. Residents with laboratory-confirmed SARS-CoV-2 infection or residents suspected of having COVID-19, based on symptoms or vital signs changes, were isolated in single rooms or housed alone in a double or triple room. The electronic medical record was used to determine daily COVID-19 trends among residents including suspected cases, laboratory-confirmed SARS-CoV-2 cases, transfers to and from acute-care hospitals, and deaths.

### Point prevalence survey testing

In addition to testing staff and residents based on symptoms or contact tracing, point prevalence survey (PPS) testing, defined as SARS-CoV-2 testing of all staff and/or all residents within a single unit, was initiated on March 25, 2020 to identify asymptomatic cases. The first PPS tested all unit A staff and residents and all unit B staff, given known cases in those units and limited testing supplies. The second PPS (April 5, 2020) included all previously negative or untested staff and residents in units A and B. Given robust symptom screening and source control among staff, the third PPS (April 14, 2020) was conducted solely among previously negative unit A residents to assess ongoing transmission as a metric for IPC efficacy. The fourth and final PPS (April 26, 2020) was conducted among previously negative unit A staff and residents.

### Laboratory testing

Nasopharyngeal specimens for SARS-CoV-2 testing were collected from staff and residents participating in PPS on units A and B, and other staff and residents were prioritized for testing by facility-wide symptom screening and contact tracing. Specimens collected by the facility were tested by the San Francisco Public Health Laboratory (SFPHL) using the Abbott RealTime SARS-CoV-2 assay (Abbott Molecular, Des Plaines, IL). Specimens collected by private providers underwent rRT-PCR, and the results were reported to facility administration. Laboratory-confirmed cases required SARS-CoV-2 detection at a cycle threshold <40.

### Whole-genome sequencing

The SARS-CoV-2–positive primary respiratory specimens were sent to CDPH for total nucleic acid extraction using the NucliSENS easyMAG system (bioMérieux, Durham, NC). Nucleic acid extracts were transferred to the University of California, San Francisco, for whole-genome sequencing (WGS) using the tiled PCR amplicon method^14^ using an Illumina NextSeq instrument (Illumina, San Diego, CA). Specimens tested at private laboratories were unavailable for WGS. We used an Amazon Web Services cloud-based computing pipeline (Amazon Web Services, Seattle, WA) for phylogenetic analysis following amplicon sequencing. The viral genome sequences were aligned using MAFFT version 7.247 software^15^ in parallel with 6,944 SARS-CoV-2 genomes from the GISAID database.^16,17^ A maximum likelihood phylogenetic tree of 6,548 unique, high-quality genomes was constructed using IQTREE v2 using the Hasegawa-Kishono-Yano substitution model.^18^ The tree and multiple sequence alignments were visualized using Geneious version 11.1.15 software (Biomatters, Auckland, New Zealand).

### Infection prevention and control interventions

The following measures were implemented in units A and B on March 22 and March 25, respectively: closure to new admissions, restriction of staff movement to other units, and controlled entry and exit of staff and residents to and from the unit. The SFDPH placed the facility on a protective order to halt admissions, and they stationed sheriffs to ensure compliance with the recommended measures in units A and B. Whenever possible, staff facility-wide were assigned to specific units and could not move between units. Ancillary services (eg, social work, physical therapy) were limited to medically essential interventions, and respiratory therapy procedures were limited to airborne infection isolation rooms. Due to PPE supply constraints, recommended PPE (ie, isolation gown, N95 respirator, gloves, and eye protection with face shield or reusable goggles) was only used by staff when directly caring for residents with suspected or confirmed SARS-CoV-2 infection. On March 26, universal surgical face masking was instituted for all unit A and B staff. IPC teams, comprised of facility administration and infection preventionists, trained staff in the appropriate use of recommended PPE, donning and doffing practices, hand hygiene, and environmental cleaning and disinfection. By April 20, IPC training was completed facility-wide and adherence monitoring continued weekly thereafter.

Beginning March 31, universal surgical face masking was instituted for all staff facility-wide. Recommended PPE was used for all clinical encounters on units A and B, regardless of resident COVID-19 status. Given critically limited PPE supplies, practices were implemented for extended use of surgical masks and extended use and reuse of respirators and face shields. Residents facility-wide were encouraged to wear cloth face coverings provided by the facility if they had no medical contraindications.

## Results

From March 22 to May 4, 2020, in a facility of 1,704 staff and 718 residents, 725 staff and residents were tested through PPSs, symptom-based testing, or contact tracing. This testing included all staff and residents in units A (n = 162) and B (n = 153), and staff and residents from other units (n = 410) who were symptomatic or were known close contacts of a person with laboratory-confirmed SARS-CoV-2 infection. In total, 512 tests were administered for testing based on contact tracing or symptom screening; 751 tests were administered for serial PPS. Of 725 persons tested, 21 (3%) were positive for SARS-CoV-2: 16 staff and 5 residents (Fig. 1).

**Fig. 1:**
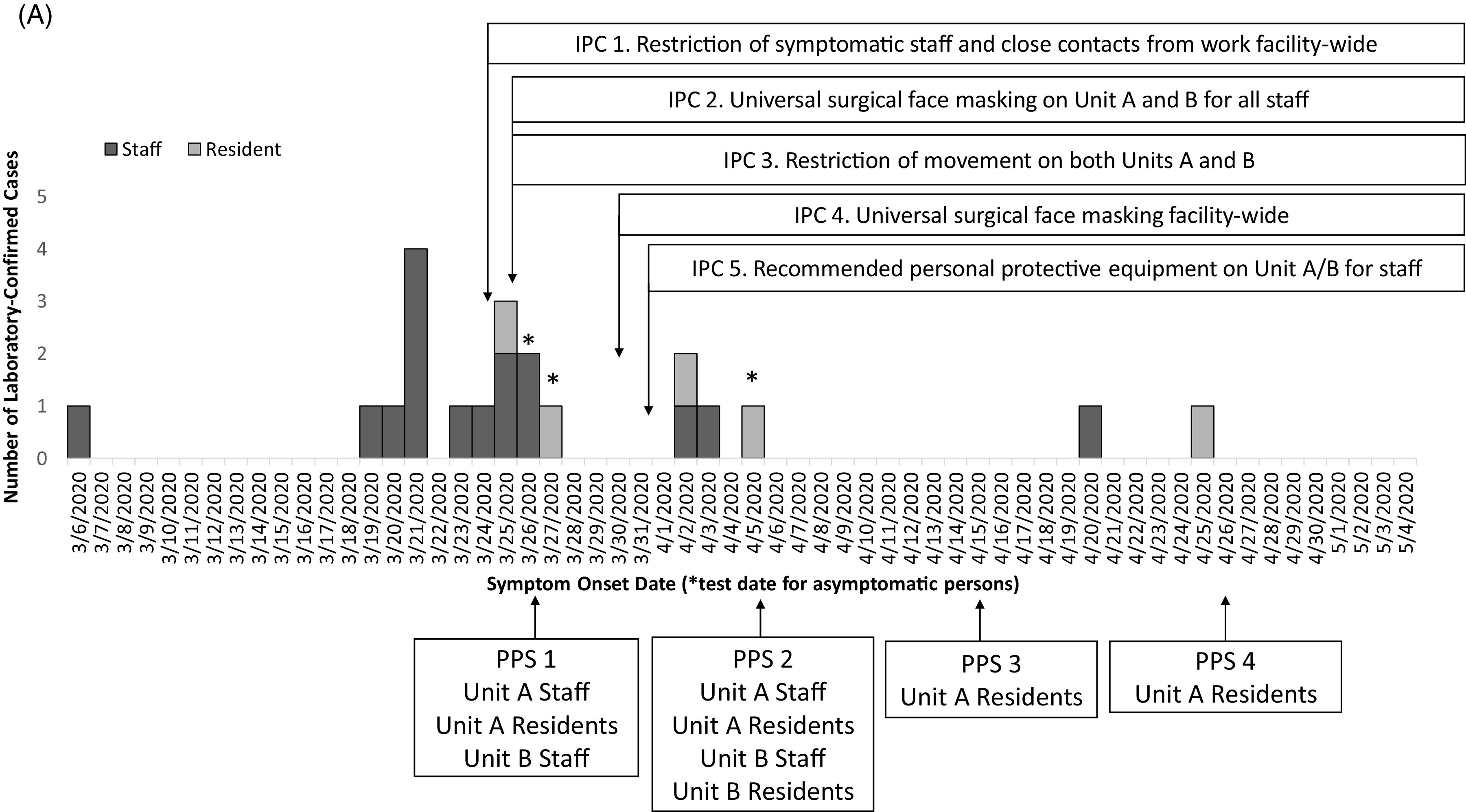
**A.** Epidemic curve of laboratory-confirmed COVID-19 cases with a timeline of the infection prevention and control (IPC) interventions and point prevalence surveys (PPS). Confirmed cases among staff (dark gray, n = 16) and residents (pale gray, n = 5) in the facility are plotted by symptom onset date or by test date (*), among 501 staff and 224 residents tested during this outbreak response. The *x*-axis also provides a timeline of the outbreak response in by test date if asymptomatic (*), IPC interventions and PPS from March 6 to May 4, 2020.

Overall, 17 cases (14 staff and 3 residents) were identified from facility-wide contact tracing or symptom screening (Table 1). During the 4 PPSs, 4 of 21 cases (19%) were identified during PPS 1 (2 staff and 1 resident) and PPS 2 (1 resident) (Table 1). No additional cases were identified in PPS 3 or PPS 4. No COVID-19–associated deaths occurred during the investigation. Fifteen cases were identified before intensive IPC implementation (ie, universal surgical face masking and recommended PPE use in units A and B), 4 cases were identified the following week, and only 2 additional cases were identified during the subsequent month (Fig. 1).

**Table 1.** Fraction and percent of cases in residents and staff in the facility identified by facility-wide targeted testing^a^ or point prevalence surveys (PPS) on Units A and B, March 22–May 4, 2020

Unit/Location	Population	Targeted Testing 1 (Mar 16–26, 2020)	PPS 1 (began Mar 26, 2020)	Targeted Testing 2 (Mar 27–Apr 5, 2020)	PPS 2 (began Apr 5, 2020)	Targeted Testing 3 (Apr 6–Apr 14, 2020)	PPS 3 (began Apr 14, 2020)	Targeted Testing 4 (Apr 15–26, 2020)	PPS 4 (began Apr 26, 2020)	Targeted Testing 5 (Apr 27–May 4, 2020)	Total Cases Identified	Unique Persons Tested
		No. cases identified/No. persons tested (% positive)										
Unit A	Staff	4/28 (14%)	2/103 (2%)	2/7 (29%)	0/99 (0%)	0/3 (0%)	-	0/1 (0%)	0/99 (0%)	-	8	107
	Residents	1/5 (20%)	1/54 (2%)	1/4 (25%)	1/52 (2%)	0/2 (0%)	0/51 (0%)	1/4 (25%)	0/50 (0%)	0/5 (0%)	5	55
Unit B	Staff	2/8 (25%)	0/92 (0%)	0/2 (0%)	0/92 (0%)	0/1 (0%)	-	-	-	0/4 (0%)	2	94
	Residents	0/2 (0%)	-	0/2 (0%)	0/59 (0%)	0/1 (0%)	-	0/3 (0%)	-	-	0	59
Other locations	Staff	3/35 (9%)	-	0/13 (0%)	-	2/88 (2%)	-	1/40 (3%)	-	0/127 (0%)	6	300
	Residents	0/17 (0%)	-	0/18 (0%)	-	0/15 (0%)	-	0/60 (0%)	-	0/17 (0%)	0	110
Total		-	-	-	-	-	-	-	-	-	21	725

a Targeted Testing refers to residents or staff tested based on symptoms, changes from baseline, or known contact to a laboratory-confirmed COVID-19 case.

**Fig. 1:**
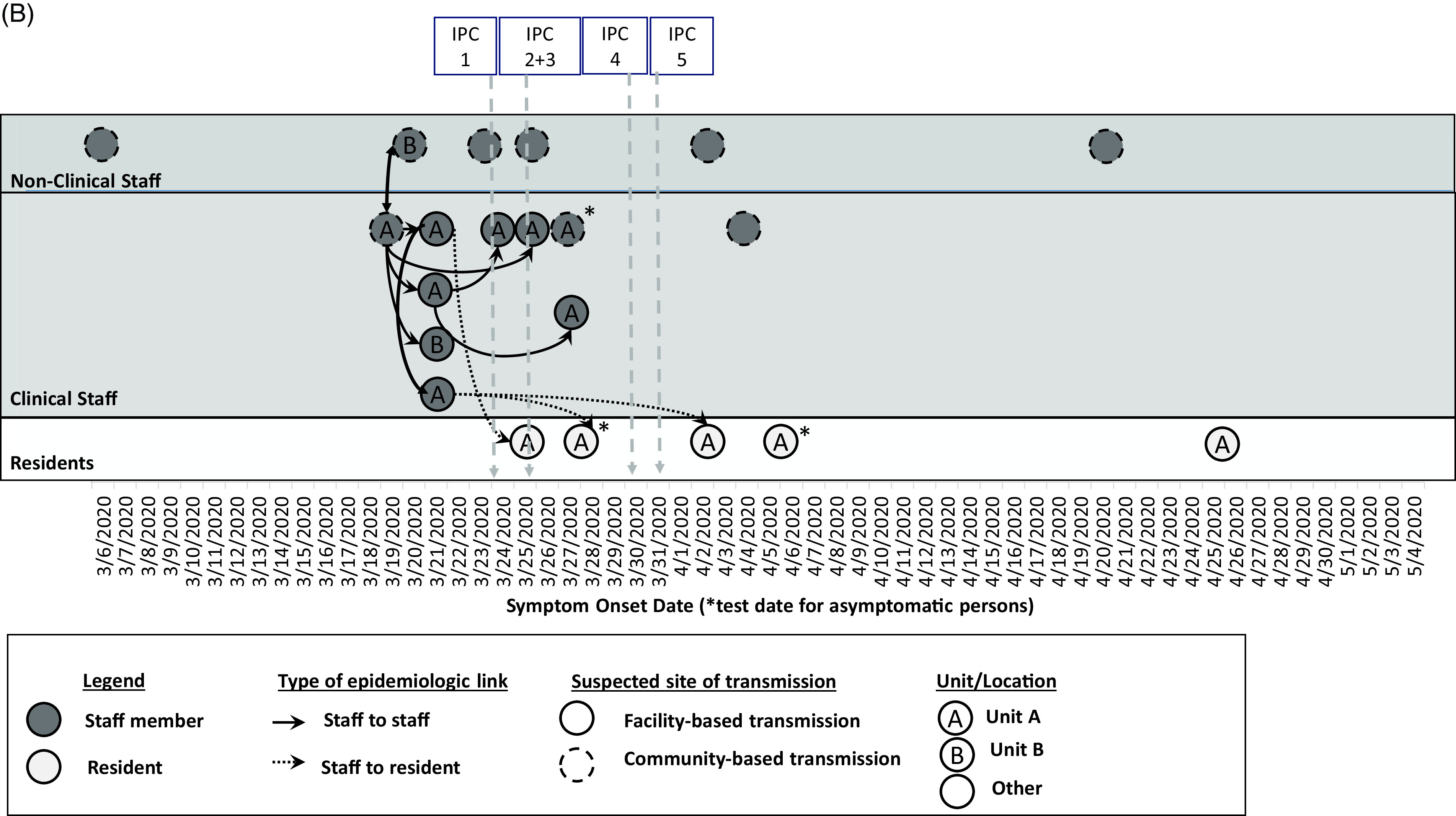
**B.** Epidemiologic linkages among laboratory-confirmed COVID-19 cases in the context of the infection prevention and control (IPC) intervention timeline (per Fig. 1A). Confirmed cases among non-clinical staff (n = 6) and clinical staff (n = 10) marked in dark gray, and residents (n = 5) marked in pale gray, are plotted by symptom onset date or by test date, if asymptomatic (*) as in Fig. 1A. Further epidemiologic data include type of epidemiologic link (staff to staff transmission in solid lines; staff to resident transmission in dotted lines), suspected site of transmission (within facility or within community), and the unit location where the case was identified. The markers at the top of the diagram refer to the timeline of the IPC interventions in Fig. 1A from March 6 to May 4, 2020.

Of 16 staff with COVID-19, there were 12 clinical care providers, 3 environmental services personnel, and 1 administrator. The earliest symptom onset date was in a staff member unassociated with units A or B who likely acquired infection from their spouse who travelled internationally. Ten staff (63%) were epidemiologically linked to unit A through work or close contacts, including the 2 staff who worked on unit B; 6 staff did not work on units A or B. Also, 9 staff were in close contact with or worked near each other (Fig. 1B); the others had no close contact to known positive staff members or residents. Among these 16 staff, 7 (44%) were tested through contact tracing, 7 (44%) were tested based on symptoms, and 2 were tested through PPS. By staff interview, we determined that the 2 staff identified by PPS had jobs at another SNF with COVID-19 cases. Of 16 SARS-CoV-2–positive staff, 2 had shortness of breath requiring emergency room care or hospitalization; both recovered.

All 5 residents with laboratory-confirmed SARS-CoV-2 infection resided on unit A; 4 (80%) were bedbound and 3 (60%) had a neurologic condition. Four residents had had no recent exposures outside the facility; the fifth resident had had an unrelated emergency room visit on April 11, 2020, and tested positive 2 weeks later. Three residents (60%) were tested based on changes from baseline: 1 had tachycardia, 1 had mental status changes, and 1 had chest pain. The other 2 residents (40%) were asymptomatic and were identified by PPS. In addition, 4 residents (80%) required emergency room care or hospitalization; all recovered and were readmitted to the facility.

**Fig. 2.**
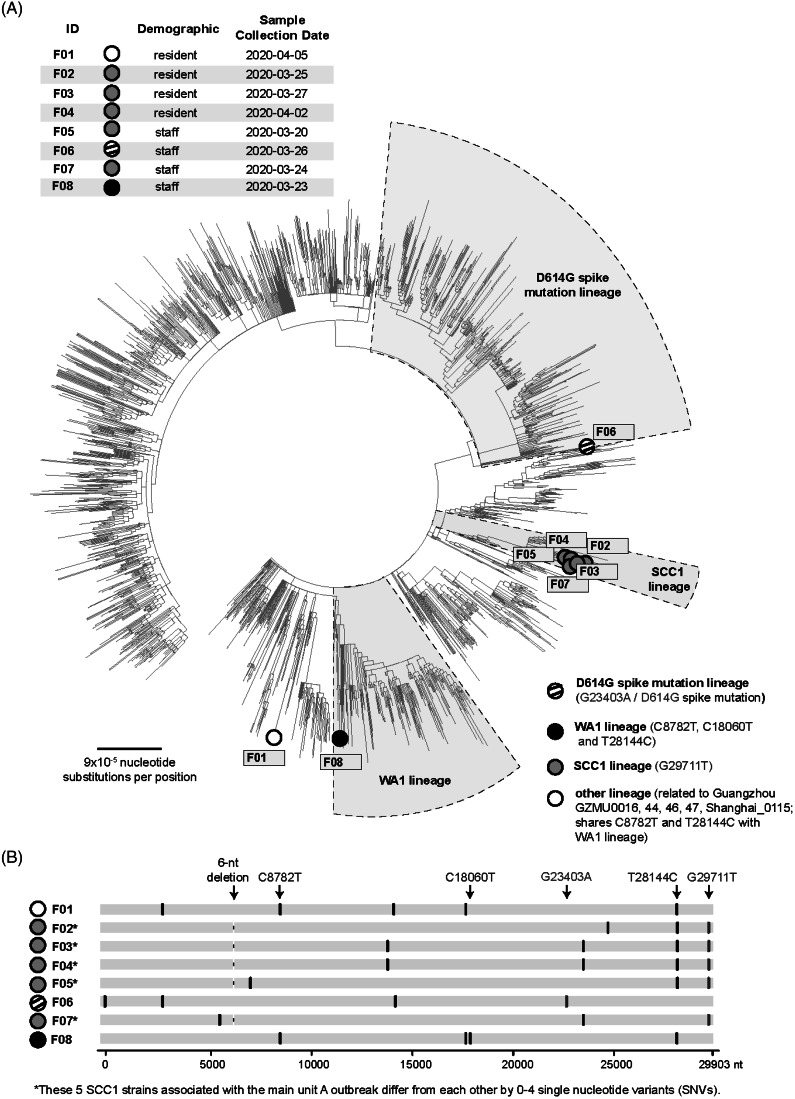
Viral lineages in the facility outbreak (n = 8) from March 22 to May 4, 2020. (A) Phylogenetic tree of the 8 facility genomes along with all SARS-CoV-2 US genomes deposited in the GISAID reference database as of May 28, 2020 and all non-US global genomes deposited as of Mar 23, 2020. The 5 strains associated with the main unit A outbreak, 3 from patients and 2 from providers, are all found to map to the SCC1 lineage. (B) Multiple sequence alignment of the 8 facility viruses. The viral genomes are aligned to the reference SARS-CoV-2 Wuhan-1 strain. Key signature SNVs relative to the reference strain are marked by lineage; other SNVs are shown in black. A 6-nt deletion is present in each of the 5 F outbreak strains. Note. F, Facility; SNVs, single nucleotide variants; WA, Washington; SCC, Santa Clara County; GISAID, Global Initiative for Sequencing of All Influenza Data (expanded to include SARS-CoV-2; nt, nucleotide).

SARS-CoV-2 virus genomes were obtained from 8 primary specimens: 4 unit A residents, 2 unit A clinical staff, 1 nonclinical staff member with close contact to a unit A staff member with COVID-19, and 1 nonclinical staff member unrelated to units A or B (Fig. 2, F01–F08). SARS-CoV-2 virus collected from March 20, 2020, through April 2, 2020, from 3 residents (F02–F04) and 2 staff [1 from unit A (F07), and the other, a close contact to unit A staff (F05)], were clustered in the northern California Santa Clara County lineage (SCC1)^19^; they differed by 0–4 single nucleotide variants (SNVs) (Fig. 2). The remaining 2 staff and 1 resident had strains from 3 distinct lineages (Fig. 2). Virus from the resident (F01) with no exposures outside the facility aligned with an early lineage from Guangzhou, China (Fig. 2). The nonclinical staff member (F08), who had not traveled, had virus from the Washington state (WA1) lineage, which had a basal position in the phylogenetic tree compared to the first COVID-19 case reported in the United States.^20^ The second staff member (F06), who also worked in a neighboring county SNF with a COVID-19 outbreak, had virus from the D614G spike mutation lineage.

## Discussion

This COVID-19 outbreak at one of the largest SNFs in the country spread rapidly among staff and then residents prior to effective IPC implementation, yet was limited to 3% of 725 unique individuals tested. Early implementation of active surveillance and contact tracing, serial PPS in units A and B, and intensified IPC measures with swift on-site public health assistance successfully limited transmission. Most viral isolates were from the SCC1 lineage, but 3 additional lineages identified in the facility suggest multiple unrelated introductions. Our investigation supports early, direct public health support for strategic testing and IPC to successfully prevent and contain outbreaks in SNFs.

Though immediate facility-wide testing to identify cases and inform IPC is ideal,^21^ supplies were insufficient to test all residents and staff during the outbreak. Instead, facility-wide symptom screening and contact tracing enabled testing of all staff and residents in units A and B (n = 315) as well as 410 staff and residents outside units A and B. This approach allowed investigators to identify individual cases in units other than A and B and to prevent associated clusters. We identified 21 (2.9%) cases of 725 unique persons tested, an exceptionally low prevalence compared to a Washington state SNF (64%)^5^ and an Illinois SNF (26%).^22^ Targeted testing identified most cases, with 17 cases (3%) identified of 512 tests administered, compared to 4 cases (0.5%) of 751 tests administered for PPS. However, given that 4 of 21 cases (19%) were identified through PPS, the importance of detecting asymptomatic infections to prevent silent spread through the facility was apparent. Routine facility-wide testing was implemented May 5, 2020, after the testing supply chain improved.

Most infections were among unit A staff who were close contacts of each other before implementation of intensive IPC, facilitating transmission to staff and residents. The reduction in new cases identified after implementing IPC measures (on-site public health assistance, universal use of face masks by staff for source control,^23^ use of recommended PPE for all resident care in units A and B, regular monitoring of IPC practices, and self-quarantine for close staff contacts of COVID-19 cases) support the efficacy of robust IPC in limiting the risk of SARS-CoV-2 transmission in this facility.

Five outbreak isolates associated with unit A were closely related in the SCC1 lineage. WGS demonstrated at least 3 additional introductions of SARS-CoV-2 into the facility; unlike a Washington state SNF, where sequenced SARS-CoV-2 strains were identical or highly related.^5^ Our results are consistent with studies reporting continual introduction of different strains into northern California during February–March 2020 associated with domestic or international travel.^24,25^ The basal WA1 strain (Fig. 2, F08) may imply even earlier SARS-CoV-2 introduction in California than expected.

Our investigation had several limitations. Although targeted testing and PPS identified most cases in units A and B and a handful of staff outside those units, the delays in facility-wide testing may have prevented early detection of asymptomatic cases in other units. Only 8 of 21 specimens (40%) underwent WGS; 2 are in process, and other specimens processed by outside labs were unavailable. It remains unclear how the resident with no exposures outside the facility (Fig. 2, F01) acquired infection with an early lineage from China; specimens from SARS-CoV-2–positive staff who cared for this resident were unavailable for sequencing, and visitor restrictions were in place. Multiple IPC measures were implemented simultaneously; thus, we cannot specify the most effective interventions. However, the decline in newly identified cases (Fig. 1) after IPC implementation supports leveraging multimodal IPC for effective control of SARS-CoV-2 transmission in SNFs, where COVID-19 outbreaks spread rapidly without early and effective containment measures.

The facility administration surmounted significant challenges during this response. The identification of 2 positive staff working at another SNF with a COVID-19 outbreak highlighted the potential for transmission between SNFs through shared staff. The facility administration began discouraging secondary employment at other facilities on April 20, 2020. Staff may be disinclined to report symptoms if work restrictions and limitations on work at outside facilities cause personal economic hardship or strain resident care; thus, the facility administration began providing extended paid sick leave for greater support. Sufficient personnel, testing, and PPE resources were difficult to obtain, slowing implementation of testing and IPC measures. Close collaboration with public health partners helped overcome these barriers, facilitating adequate PPE access and a testing turnaround time of 72 hours for residents and staff.

This report demonstrates the value of rapid, collaborative intervention after initial SARS-CoV-2 identification in a SNF, including resource-conscious testing strategies, PPS to identify asymptomatic cases and prevent undetected transmission, intensive IPC measures, and active surveillance for infections among staff and residents given continued community-based transmission. Although routine facility-wide testing was unavailable during the outbreak and resource limitations were encountered, this multimodal approach successfully limited the extent of a COVID-19 outbreak in a large SNF. The intensive IPC measures and surveillance implemented by the facility has continued throughout the course of the COVID-19 pandemic to prevent and control future outbreaks.
